# Co-administration of tyrosine kinase inhibitors with rottlerin in metastatic prostate cancer cells

**DOI:** 10.17179/excli2021-3980

**Published:** 2021-11-19

**Authors:** Wojciech A. Cieslikowski, Tobias Haber, Slavomir Krajnak, Katharina Anic, Annette Hasenburg, René Mager, Joachim W. Thüroff, Walburgis Brenner

**Affiliations:** 1 Department of Urology and Pediatric Urology, Johannes Gutenberg University Medical Center, Langenbeckstr. 1, 55131 Mainz, Germany; 2 Department of Urology, Poznan University of Medical Sciences, 61-701 Poznan, Poland; 3 Clinic for Obstetrics and Women's Health, Johannes Gutenberg University Medical Center, Langenbeckstr. 1, 55131 Mainz, Germany

**Keywords:** prostate cancer, PC-3, LNCaP, rottlerin, Tyrosine kinase inhibitors, PKCdelta

## Abstract

After prostatectomy due to prostate carcinoma, patients often develop metastases. Although prostate cancer is susceptible to hormonal manipulation, many patients become castration-resistant. Therefore, new therapies are the focus of investigations. We analyzed the effect of the tyrosine kinase inhibitors (TKIs), sorafenib and sunitinib, in combination with rottlerin, a PKCδ inhibitor, on metastatic mechanisms in prostate carcinoma cells. LNCaP and PC-3 prostate carcinoma cells were treated with sorafenib or sunitinib alone at various concentrations (1-20 µM) or in combination with rottlerin (10 µM) for 24 h. Then, cell toxicity (MTT test) and cell proliferation (BrdU incorporation assay) were quantified. The study demonstrated a dose-dependent inhibitory effect of sorafenib and sunitinib on PC-3 and LNCaP cell activity and proliferation. Both agents showed significantly stronger cytotoxic effects in LNCaP cells. At the highest concentrations, sorafenib and sunitinib inhibited the viability of LNCaP cells up to 2 % and 31 %, respectively, and the viability of PC-3 cell line up to 20 % and 43 %, respectively. The proliferation of both cell lines was significantly stronger inhibited by sorafenib than by sunitinib. In LNCaP cells, sorafenib and sunitinib at the highest concentrations inhibited cell proliferation up to 46 % and 49 %, respectively, and the proliferation of PC-3 line up to 40 % and 47 %, respectively. Rottlerin reduced the viability and proliferation of PC3 cells to 81 % and 42 %, whereas the viability and proliferation of LNCaP cells were reduced to 25 % and 57 %, respectively. Sorafenib and sunitinib at low concentrations partly neutralized the inhibitory effect of rottlerin on cell viability and proliferation. On the other hand, in PC-3 cells, rottlerin reduced the inhibitory effects of sorafenib and sunitinib at the highest concentrations on cell viability from 20 % to 30 % and from 43 % to 61 %, respectively. An additive effect on cell activity was observed after treating LNCaP cells with both sunitinib at high concentrations and rottlerin. This combination increased the cytotoxic effect from 31 % to 13 % at the highest sunitinib concentration. Our results showed that monotherapy with sorafenib was the most efficient in both PCa cell lines. A marginally additive effect of rottlerin was only observed in LNCaP cells treated with sunitinib at a high concentration. Sorafenib and sunitinib reduced cell migration in PC-3 cells to 10 % and 32 % of untreated cells, respectively. Co-treatment with sorafenib/sunitinib and rottlerin did not result in a significantly stronger anti-migratory effect than the treatment with each TKI alone. Given the strong cytotoxic effect of TKIs, especially sorafenib, on LNCaP cells, the results of the migration assay in this line were severely biased and not considered in the analysis. Unlike in other malignancies, combination therapy with TKI and rottlerin seems not beneficial in prostate cancer. More promising seems to be monotherapy with rottlerin, but further studies are needed to confirm this observation.

## Introduction

Prostate cancer (PCa) is one of the major medical problems the male population faces. Prostate cancer is the most frequent cancer among males in Europe (Arnold et al., 2015[3]). Unfortunately, about 20 % of all newly diagnosed PCa cases present as metastatic disease and many others progress to metastases despite surgical treatment, chemotherapy or hormone therapy (androgen ablation). Because of the poor prognosis in prostate cancer patients, there is an urgent need for novel treatments in men with this malignancy (Maitland, 2021[29]).

Targeted therapies with tyrosine kinase inhibitors (TKIs), sorafenib and sunitinib, seem a promising option for prostate cancer patients (Gallick et al., 2012[11]). Sunitinib, an oral TKI with antiangiogenic and antiproliferative activity, is a broad-spectrum multi-targeted tyrosine kinase inhibitor targeting vascular endothelial growth factor receptor (VEGFR-1, VEGFR-2, VEGFR-3), platelet-derived growth factor receptors (PDGFR-A, PDGFR-B), basic fibroblast growth factor receptor (bFGFR), stem cell growth factor receptor (c-Kit), FMS-like tyrosine kinase 3 (FLT-3), RET tyrosine kinase receptor, and colony stimulating factor 1 receptor (CSF-1-R) (Chow and Eckhardt, 2007[9]). Sorafenib targets VEGFR-2, VEGFR-3, PDGFR, and multiple kinases, such as RAF, c-Kit and c-Ret. Both agents interact with tumor signaling, inhibiting angiogenesis and tumor cell proliferation (Faivre et al., 2007[10]; Oh et al., 2012[34]). 

However, the success rate of prostate cancer therapy using TKIs is limited, and some patients develop resistance (Aragon-Ching et al., 2009[2]). In a phase II study of sorafenib in castration-resistant prostate cancer (CRPC), therapeutic activities were observed in the RECIST criteria, such as preventing radiologic progression and regression of bone metastases. However, no decline in PSA was documented. Moreover, a response or disease stabilization in chemotherapy naïve CRPC patients was observed only in 47 % of those treated with sorafenib combined with bicalutamide, an antiandrogen agent (Beardsley et al., 2012[5]). Sorafenib is currently evaluated in studies involving docetaxel refractory PCa patients, as in some cases, it could overcome chemotherapy resistance (Meyer et al., 2014[32]).

Initial phase I/II clinical studies found that sunitinib is a promising agent in treating CRPC patients (Zurita et al., 2012[42]). However, a phase III study investigating sunitinib plus prednisone in patients with a metastatic CRPC after the failure of docetaxel chemotherapy (SUN 1120), with OS as the primary endpoint, was recently prematurely discontinued due to the lack of efficacy (clinicaltrials.gov, 2020). Although targeted therapies of CRPC have clear benefits, their success rate is not sufficient. Several mechanisms have been uncovered explaining the resistance to sunitinib or sorafenib (Krebs et al., 2020[23]). New strategies concentrate on combining already approved medications or finding new agents to be combined with TKIs (Mardjuadi et al., 2012[31]; Lu et al., 2021[26]; Spetsieris et al., 2021[38]). 

The protein kinase C (PKC) family has been discussed as a target for anticancer therapy (Hofmann, 2004[13]). PKC is highly expressed in prostate cancer (Mukherjee et al., 2009[33]). Regulating several intracellular signaling pathways, PKC has been linked to the carcinogenesis of many malignancies, including prostate cancer (Ratnayake et al., 2021[35]). Within the family of PKC isoforms, PKCδ plays a critical role in regulating apoptosis and tumor progression (Yamada et al., 2021[40]). PKCδ is a serine-threonine kinase with opposing functions that depend on its localization, tyrosine phosphorylation and the presence of other pro- and anti-apoptotic signaling molecules. Activating MAPK and AKT signaling pathways, PKCδ has an oncogenic function, inducing cell proliferation and inhibiting apoptosis (Jane et al. 2006[17]). Localized in the nucleus, it is involved in the initiation of apoptosis. In this context, PKCδ has been implicated in tumor suppression and the survival of different cancers (Basu and Pal, 2010[4], Halvorsen et al., 2020[12]). PKCδ not only contributes to apoptosis and cell proliferation but also regulates cell migration via integrin β1 and focal adhesion kinase (FAK) (Brenner et al., 2008[8]), leading to cancer progression. This implicates PKCδ as a potential target for anticancer therapy. Since PKCδ activates AKT and MAPK signaling pathways downstream of the targets of TKI (Allen-Petersen et al., 2014[1]), an additional inhibitory effect can be expected after simultaneous inhibition of this kinase, as already suggested in glioma cells (Jane et al., 2006[17]). A known inhibitor of PKCδ is rottlerin (mallotoxin). Rottlerin is a 5,7-dihydroxy-2,2-dimethyl-6-(2,4,6-trihydroxy-3-methyl-5-acetylbenzyl)8-cinnamoyl-1,2-chromene, extracted from *Mallotus philippinensis*. Although rottlerin is described as a PKCδ-specific inhibitor, it is known to inhibit also several other signaling molecules and cellular mechanisms (Maioli et al., 2012[28], 2018[27]). As early as in the 19^th^ century, rottlerin has been known as a therapeutic agent used as an antihelminth and laxative (Soltoff, 2007[37]). Rottlerin-induced early autophagy is mainly dependent on the induction of autophagosomes, conversion of LC3-I - LC3-II, expression of Atg12 and Beclin-1 and inhibition of Bcl-2, Bcl-xL, XIAP and cIAP-1. Rottlerin induces apoptosis by inhibiting PI3K/AKT/ mTOR and AMPK pathways and activating caspases (Kumar et al., 2014[24]).

The aim of this study was to investigate whether simultaneous pharmacological inhibition of TKI pathways and administration of rottlerin have additive antiproliferative and cytotoxic effects on metastatic prostate cancer cells. We hypothesized that this combination might synergistically block prostate cancer cell proliferation and migration.

## Materials and Methods

### Cell lines

All experiments were conducted with the use of human prostate carcinoma cell lines, LNCaP and PC-3. Both cell lines originated from a high-grade prostate cell carcinoma (Horoszewicz et al., 1980[14]; Kaighn et al., 1978[19]) and were obtained from the American Type Culture Collection (Manassas, VA, USA). The LNCaP cells were isolated by Horoszewicz et al. (1983[15]) from a needle aspiration biopsy of the left supraclavicular lymph node of a 50-year-old Caucasian male with a confirmed diagnosis of metastatic prostate carcinoma. These cells are responsive to 5-alpha-dihydrotestosterone (growth modulation and acid phosphatase production). The PC-3 line was derived by Kaighn et al. (1979[20]) from bone metastasis of a grade IV prostatic adenocarcinoma in a 62-year-old Caucasian male. The cells exhibit low acid phosphatase and testosterone-5-alpha reductase activities.

The cells were cultured in RPMI-1640 with L-glutamine (Gibco, Eggenstein, Germany), supplemented with 10 % fetal calf serum (FCS), 2.5 % Hepes and 1 % penicillin/streptomycin. The cultures were grown and maintained at 37 °C in a humidified atmosphere with 95 % air/5 % CO_2_ and subcultured every 4 to 7 days by treatment with Trypsin-EDTA. Cell concentrations in trypsinized samples were determined in a Neubauer chamber.

### Cell viability assay

The cytotoxic effects of rottlerin and TKIs were determined using an MTT assay (Toxicology Assay Kit MTT, Sigma-Aldrich, Inc. St. Louis, U.S.A.) (Brenner et al., 2010[6]). The yellow MTT [3-(4,5-dimethyltiazol-2-yl)2,5-diphenyl tetrazolium bromide] was reduced to a blue formazan product by mitochondrial activity. 5 x 10^3^ cells per well were seeded in a 96-well plate and incubated for 24 h at 37 °C with 5 % CO_2_. Cells were incubated in serum-free medium for 24 h and afterwards treated with sorafenib and sunitinib (both LC Laboratories, Woburn, U.S.A.) in concentrations of 0 μM (control), 1 μM, 2.5 μM, 5 μM, 10 µM and 20 μM, alone or in combination with 10 μM of rottlerin (Calbiochem, Darmstadt, Germany) in serum-free culture medium for 24 h. Subsequently, the medium was replaced with 100 μl of MTT working solution (0.5 mg/ml in phenol red-free medium), and the plate was incubated for two hours. To dissolve the colored crystalized product, 100 μl of Solubisation Solution (from MTT test kit) was added to each well, the plate was wrapped in dark and shaken for at least one hour at 500 rpm. The intensity of blue staining was measured spectrophotometrically at 570 nm wavelength (reference wavelength 690 nm).

### Cell proliferation assay

The cell proliferation assay was performed with ELISA BrdU (bromodeoxyuridine) colorimetric assay (Roche Diagnostics GmbH, Mannheim, Germany) (Brenner et al., 2004[7]). The cells were seeded in a 96-well plate as described above, incubated in serum-free medium for 24 h and exposed to the same concentrations (0-20 μM) of sunitinib and sorafenib with/without rottlerin (10 μM) for another 24 h. After incubation, 10 μl of BrdU solution was added per well and incubated for 2 h at 37°C. After removing the culture medium, the cells were fixed, and the DNA was denaturated in one step by adding fixDenat solution. Incorporated BrdU was detected by an anti-BrdU-POD antibody. The immune complex was detected by a substrate reaction and quantified by measuring the absorbance at 450 nm wavelength (reference wavelength 690 nm) in an ELISA-reader.

### Migration assay

Chemotactical cell migration was quantified in a Boyden chemotaxis chamber (Costar, Bodenheim, Germany). Before starting the experiment, the cells were cultured in a serum-free medium for 24 h. Subsequently, the cells were treated with sunitinib (5 μM) or sorafenib (5 μM) with/without 10 μM of rottlerin for 24 h. A chemotaxis chamber with a porous polycarbonate membrane (pore diameter 8 μM) was divided into 48 wells to obtain an invasion unit with a surface of ~7.8 mm^2^. In the lower part of the chamber, chemotaxin (fibronectin, 10 μg/ml) was dissolved in an FCS-free medium. The upper part of the chamber was loaded with 50 μl of cell suspension (3 x 10^5^ cells/ml), and the chamber was incubated for 16 h at 37 °C in a 5 % CO_2_ atmosphere in the presence of the agents. Subsequently, the cells on the upper side of the polycarbonate membrane were removed. The migrated cells were fixed in methanol, stained with hemacolor (Merck) and counted in an area of 2.5 cm^2^ (10 views per well) using a test raster ocular (Zeiss, 400-fold magnification) (Schneider et al., 2011[36]; Joeckel et al., 2014[18]).

### Statistical analysis

Each experiment was performed in quadruplicate and repeated in three independent series. The results were presented as percentages of the control (untreated cells). The statistical characteristics of results were presented as medians and interquartile ranges. The results of the tests in cultures treated with various concentrations of TKIs with/without rottlerin were compared by means of the Mann-Whitney U-test. All calculations were carried out with IBM SPSS 23 (IBM, Frankfurt, Germany), with the significance level set at p≤0.05.

## Results

### Effect of TKI and rottlerin on cell viability

**Figure 1:**
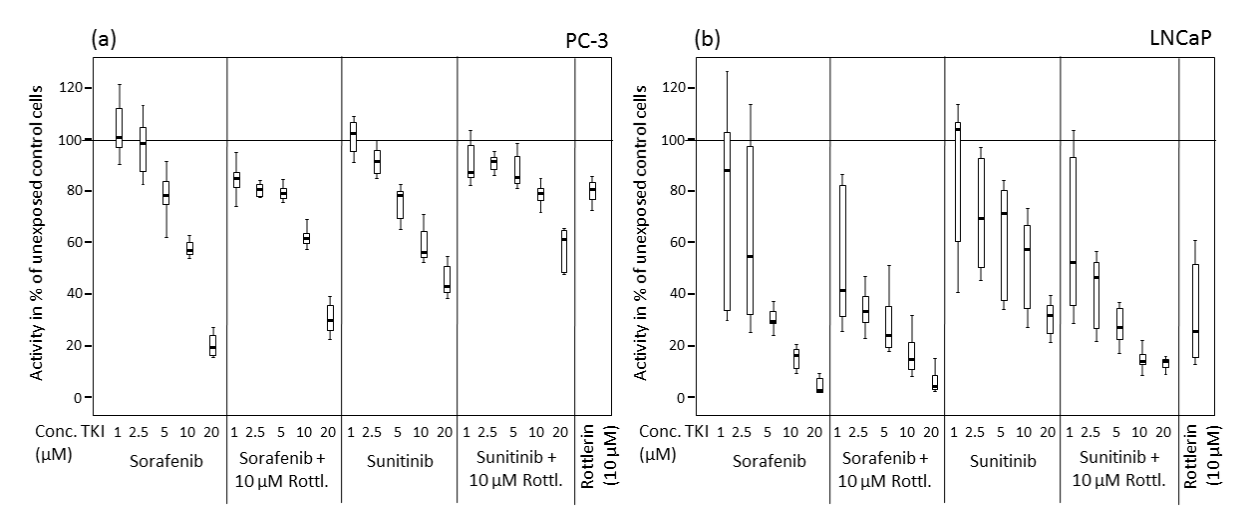
Viability of prostate cancer cells exposed to various concentrations of sorafenib and sunitinib with/without rottlerin (10 µM), determined in an MTT assay. (a) PC-3 cell line; (b) LNCaP cell line.

After treatment with sorafenib and sunitinib alone or combined with rottlerin, cell viability was reduced in a concentration-dependent manner (Table 1(Tab. 1), Figure 1(Fig. 1)). In PC-3 cells, especially at low concentrations, the TKI/rottlerin combination treatment had a significantly higher inhibitory effect on cell viability than with each TKI alone (p<0.005). In contrast, at a higher concentration of TKI, this effect reversed. In LNCaP cells, the viability inhibition after combination treatment with sunitinib and rottlerin was significantly stronger compared with sunitinib alone (p<0.002), and at high sunitinib concentration also compared with rottlerin alone (p<0.02). After treatment of LNCaP cells with a low concentration of sunitinib in combination with rottlerin, the inhibitory effect of rottlerin was partly abolished. At high concentrations, the cytotoxic effect of sorafenib on both cell lines was significantly stronger than the effect of sunitinib (p<0.001; Table 1(Tab. 1), Figure 1(Fig. 1)). The cytotoxic effect of rottlerin alone was significantly stronger in LNCaP cells than in the PC-3 line (p<0.001; Table 1(Tab. 1)). 

### Effect of TKI and rottlerin on cell proliferation

**Figure 2:**
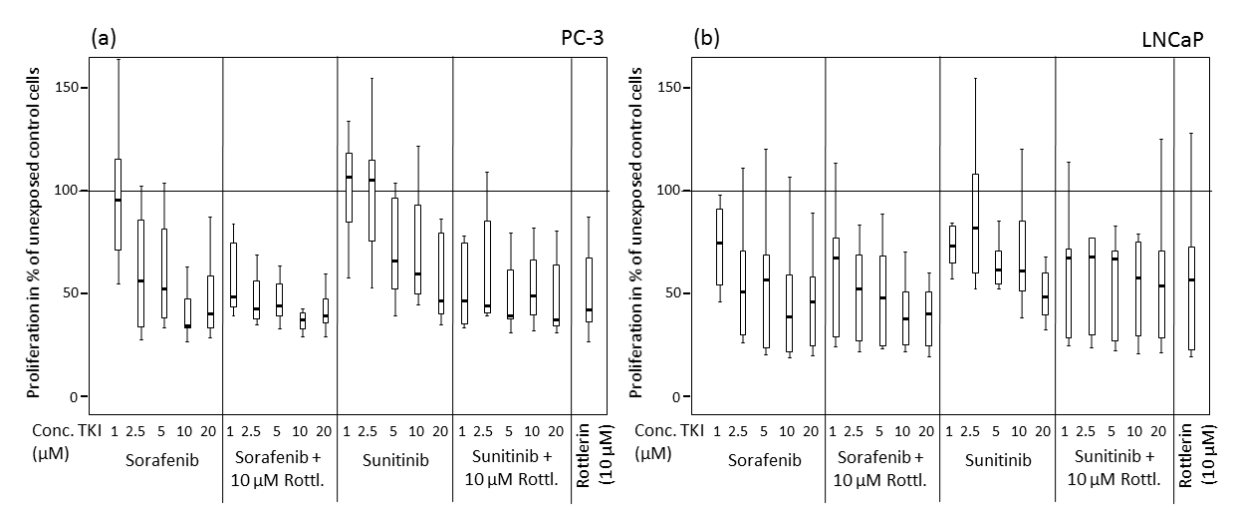
Proliferation of prostate cancer cells exposed to various concentrations of sorafenib and sunitinib with/without rottlerin (10 µM), determined by BrdU incorporation. (a) PC-3 cell line; (b) LNCaP cell line.

Similar to the cell viability, the cell proliferation after treatment with sorafenib and sunitinib alone or in combination with rottlerin was reduced in a concentration-dependent manner (Table 2(Tab. 2), Figure 2(Fig. 2)). At 2.5 µM and 10 µM, sorafenib produced a significantly stronger antiproliferative effect in both cell lines than sunitinib (p<0.004). Treatment with rottlerin potentiated the antiproliferative effect of sorafenib solely in PC-3 cells at 1 µM (p=0.001), whereas co-treatment with sunitinib at low concentration together with rottlerin resulted in a significantly weaker proliferation than the treatment with sunitinib alone (p<0.01). However, cell proliferation after co-treatment with sunitinib and rottlerin was not inhibited stronger than after the treatment with rottlerin alone.

### Effect of TKI and rottlerin on cell migration

**Figure 3:**
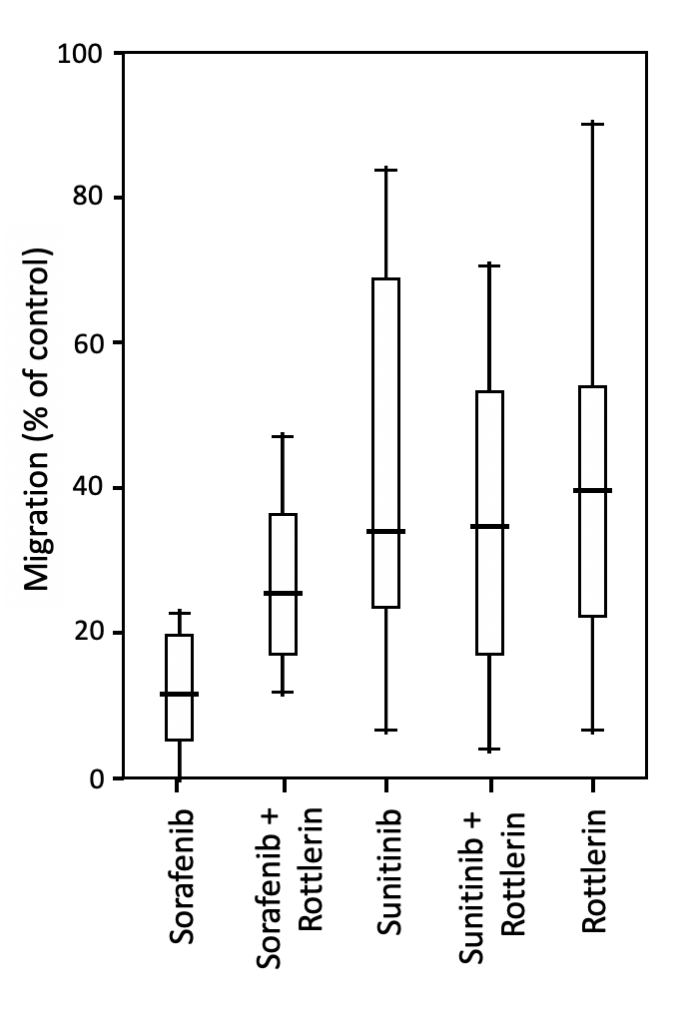
Migration of PC-3 prostate cancer cells exposed to sorafenib and sunitinib (5 µM each) with/without rottlerin (10 µM), determined in a Boyden chamber with fibronectin (10 µM) as chemotaxin.

Sorafenib and sunitinib reduced the migration of PC-3 cells to 10 % and 32 % of untreated cells, respectively, with the effect of sorafenib being significantly stronger than that of sunitinib. Treatment with rottlerin alone reduced cell migration in the PC-3 line to 37 %. Co-treatment with sorafenib/ sunitinib and rottlerin did not result in a significantly stronger anti-migratory effect than the treatment with each TKI alone (Table 3(Tab. 3); Figure 3(Fig. 3)). Given the strong cytotoxic effect of TKIs, especially sorafenib, on LNCaP cells, the results of the migration assay in this line were severely biased and are not shown.

## Discussion

This study demonstrated that both TKIs, sorafenib and sunitinib, reduced the viability and proliferation of prostate cancer cells PC-3 and LNCaP in a concentration-dependent manner, as well as inhibited the migration of the PC-3 cells. While the treatment with rottlerin also contributed to a decrease in the cell viability, proliferation and migration, with a few exceptions (viability of LNCaP cells treated with sunitinib at higher concentrations), no significant additive effect of a TKI plus rottlerin combination on the cytotoxic, antiproliferative and anti-migratory activity was observed. The cytotoxic, antiproliferative and anti-migratory effects of sorafenib tended to be stronger than those of sunitinib, and both TKIs were more effective against the PC-3 line than in LNCaP prostate cancer cells.

To the best of our knowledge, a potential additive effect of rottlerin and TKIs on cancer cells was a subject of only one published study. Jane et al. (2006[17]) analyzed the effects of sorafenib and rottlerin on malignant glioma cell lines using proliferation assays, apoptosis induction studies and Western immunoblot analysis. Sorafenib and rottlerin produced antiproliferative effects in all cell lines when used as single agents, and even more pronounced growth inhibition was observed after the exposure of glioma cells to sorafenib plus rottlerin combination. Moreover, the addition of rottlerin potentiated the proapoptotic effect of sorafenib. The authors concluded that the addition of rottlerin enhanced the antiproliferative effect of sorafenib on glioma cells, which warrants further research on the combinations of signaling inhibitors as an anticancer treatment modality (Jane et al., 2006[17]).

Regarding the potential application of rottlerin as an adjuvant treatment in prostate cancer, Hsu et al. (2012[16]) analyzed the effect of combination treatment with this agent and some other anticancer drugs on apoptosis in HRPC PC-3 line using FACScan flow cytometric analysis of PI staining. Rottlerin was shown to potentiate camptothecin-induced DNA fragmentation at the S phase and ATM phosphorylation, which correlated with the apoptosis of prostate cancer cells (Hsu et al., 2012[16]). 

Interestingly, our present study did not confirm those promising findings, as the addition of rottlerin did not potentiate the cytotoxic and antiproliferative effects of sorafenib and sunitinib in prostate cancer cells. This observation is consistent with the results of the previously mentioned study conducted by Hsu et al. (2012[16]). Other than for camptothecin, no additive effect of rottlerin against prostate cancer cells was observed in etoposide-, doxorubicin- and vincristine-treated cultures, and rottlerin even caused a significant decrease in docetaxel-induced apoptotic effect (Hsu et al., 2012[16]).

The abovementioned discrepancies in the anticancer activity of rottlerin might be associated with the pleiotropic character of this compound. While rottlerin was initially classified as a PKCδ inhibitor, a growing body of evidence suggests that instead of interacting with a single well-defined target, this molecule acts via numerous biochemical and molecular mechanisms (Maioli et al., 2012[28]). Indeed, in the study conducted by Jane et al. (2006[17]), Western blot analysis demonstrated that sorafenib affected phosphorylation of ERK and AKT kinases in glioma cells, and this effect was further potentiated by rottlerin. Hence, this agent acted in a PKCδ-independent mechanism (Jane et al., 2006[17]). Also, in multiple other studies involving rottlerin, this agent produced its anticancer effects via different mechanisms than PKCδ inhibition (Maioli et al., 2012[28]). The pleiotropic character of rottlerin was also confirmed in several studies involving prostate cancer cells. Similar to our present experiment, in the study conducted by Kharait et al. (2006[21]), rottlerin reduced the migration and invasion of prostate cancer cells PC-3 (and also DU145); according to the authors of that study, the anticancer effects of rottlerin were caused by the inhibition of PKCδ expression. In turn, according to Kumar et al. (2014[24]), rottlerin induced autophagy and apoptosis in prostate cancer stem cells via the PI3K/AKT/mTOR signaling pathway. Lu et al. (2014[25]) demonstrated that rottlerin induced Wnt co-receptor LRP6 degradation and suppressed both Wnt/β-catenin and mTORC1 signaling pathways in prostate cancer cells PC-3 and DU145. Finally, in a most recent study, Zheng et al. (2018[41]) found that rottlerin inhibited cell growth, migration and invasion, and induced apoptosis in PC-3 and DU145 prostate cancer cells through the downregulation of EZH2 and H3K27me3. Unfortunately, unlike the experiments mentioned above, our study did not include targeted molecular analyses, so we could not identify the exact mechanism behind the antiproliferative, cytotoxic and anti-migratory effect of rottlerin applied alone or in combination with a TKI.

Interestingly, in our present study, both TKIs were more efficient in LNCaP cells than in the PC-3 line. The main difference between these two cell lines is that LNCaP cells, expressing androgen receptor (AR), are androgen-sensitive, whereas PC-3 cells are androgen-insensitive. Perhaps the phenomenon observed in our study was associated with AR expression in LNCaP cells, which is regulated by the PI3K/AKT pathway in normal and malignant epithelial cells. A growing body of evidence suggests that key factors of the PI3K/AKT/mTOR pathway may directly regulate the expression and transcriptional activity of AR. Specifically, it has been demonstrated that AR phosphorylation and activation by AKT occur predominantly at low androgen concentrations, suggesting a significant role of AKT in stimulating cell growth following castration. One study revealed that the inhibition of PI3K/AKT pathway by LY294002 decreased dihydrotestosterone-induced expression of AR in LNCaP cells, and the expression of a dominant-negative AKT blocked AR expression (Manin et al., 2002[30]). Consequently, also the inhibition of AKT by TKIs and rottlerin could be reflected by reduced activity of AR and androgen-dependent cells. Furthermore, Sumitomo et al. (2004[39]) showed that LNCaP cells suspended in a medium containing 10 % FCS expressed a higher level of PKC protein compared with PC-3 cells. This could be another reason behind the greater susceptibility of LNCaP cells to TKIs and rottlerin documented in our study. Moreover, a recent study showed that PC-3 cells had increased NADPH oxidase activity and HIF-1α levels, which was partly responsible for their angiogenic activity, and thus, invasiveness (Kim et al., 2011[22]).

We are well aware of some potential limitations of this study. As mentioned above, we analyzed the effects of TKIs and rottlerin without insight into their underlying molecular mechanisms. Meanwhile, identifying the molecular background in a given experimental setting seems crucial given the pleiotropic character of rottlerin. Further, given the strong cytotoxic effect of TKIs on LNCaP cells, the results of the migration assay in this line were severely biased and could not be considered in the analysis.

## Conclusions

After prostatectomy due to prostate carcinoma, many patients develop metastases. Therefore, new therapies are being investigated. Targeted therapies with TKIs are promising treatments for prostate cancer. In this study, the effects of two TKIs, sorafenib and sunitinib, on metastatic mechanisms in prostate cancer cell lines were investigated. Since in other tumor types, rottlerin enhanced the antiproliferative effects of sorafenib through PKCδ inhibition, a co-administration of rottlerin with sorafenib or sunitinib was investigated. The cytotoxic effect of sorafenib on cell viability was enhanced by rottlerin only at the lowest concentration. Sunitinib treatment resulted in a lesser cytotoxic effect. However, co-administration of rottlerin potentiated this effect. The inhibition of the proliferation by either sorafenib or sunitinib was not influenced by rottlerin. Co-treatment of PC-3 cells with sorafenib/sunitinib and rottlerin did not result in a significantly stronger anti-migratory effect than the treatment with each TKI alone. Sorafenib produced the strongest inhibitory effect on cell proliferation, with no additive effect of rottlerin. These results point to monotherapy with sorafenib as the most effective option in preventing prostate carcinoma spread. Also, the results of monotherapy with rottlerin seems promising. However, combination therapy with a TKI and rottlerin did not produce an additional benefit. Further mechanistic studies of rottlerin are needed to fully decipher its molecular effects in metastatic prostate cancer cells.

## Declaration

### Author contributions

Conceptualization, WAC and WB; methodology, WAC; validation, WB; formal analysis, WAC; investigation, WAC; resources, JWT; data curation, WAC, WB; writing-original draft preparation, WAC; writing-review and editing, TH, RM, SK, KA, AH, JWT; supervision, JWT; project administration, WB. All authors have read and agreed to the published version of the manuscript. 

### Funding

This research received no external funding.

### Institutional Review Board Statement

Not applicable. 

### Informed Consent Statement

Not applicable.

### Conflict of interest

The authors declare no conflict of interest.

## Figures and Tables

**Table 1:**
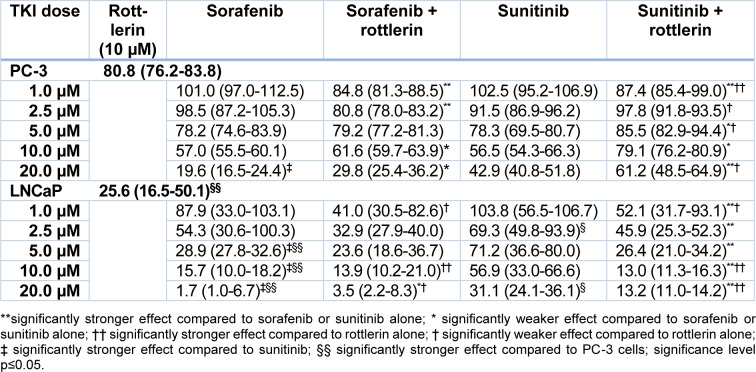
Viability of PC-3 and LNCaP prostate cancer cells exposed to various concentrations of sorafenib and sunitinib with/without rottlerin (10 µM), determined in an MTT assay. The results are presented as medians (interquartile ranges) of the activity in the percentage of unexposed control cells.

**Table 2:**
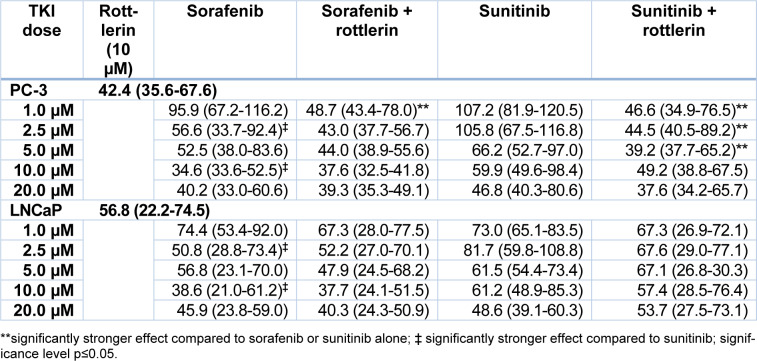
Proliferation of PC-3 and LNCaP prostate cancer cells exposed to various concentrations of sorafenib and sunitinib with/without rottlerin (10 µM), determined by BrdU incorporation. The results are presented as medians (interquartile ranges) of the activity in the percentage of unexposed control cells.

**Table 3:**
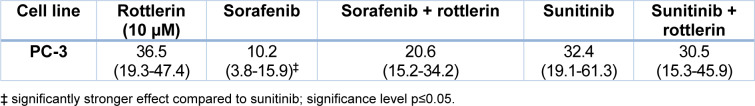
Migration of PC-3 prostate cancer cells exposed to sorafenib and sunitinib (5 µM each) with/without rottlerin (10 µM), determined in a Boyden chamber with fibronectin (10 µM) as chemotaxin. The results presented as medians (interquartile ranges) of the activity in the percentage of unexposed control cells.
